# Study of the Role of Dopamine Receptors in Streptozotocin-Induced Depressive-Like Behavior Using the Forced Swim Test Model

**DOI:** 10.22086/gmj.v0i0.954

**Published:** 2018-04-01

**Authors:** Afshin Roostaei, Gholamhassan Vaezi, Mohammad Nasehi, Ali Haeri-Rohani, Mohammad-Reza Zarrindast

**Affiliations:** ^1^Department of Biology, Damghan Branch, Islamic Azad University, Damghan, Iran; ^2^Cognitive and Neuroscience Research Center (CNRC), Tehran Medical Sciences Branch, Islamic Azad University, Tehran, Iran; ^3^Department of Animal Biology, School of Biology, University College of Science, University of Tehran, Tehran, Iran; ^4^Department of Pharmacology, School of Medicine, Tehran University of Medical Sciences, Tehran, Iran; ^5^Iranian National Center for Addiction Studies, Tehran University of Medical Sciences, Tehran, Iran; ^6^Institute for Cognitive Science Studies (ICSS), Tehran, Iran

**Keywords:** Streptozotocin, Depression, Dopamine Receptors

## Abstract

**Background:**

Diabetes is one of the most common endocrine diseases characterized by hyperglycemia. It is caused by an absolute or relative insulin deficiency or an insulin function deficiency. It is one of the major risk factors of depression, with the rate of depression in diabetic patients amounting to as high as 30%. This study examined the role of dopamine receptors in streptozotocin (STZ)-induced depressive-like behavior using the forced swim test (FST).

**Materials and Methods:**

This study was performed on 56 Wistar male rats. STZ at doses of 30 and 60 mg/kg body weight was administered via intraperitoneal (IP) route to induce diabetes and depression in rats. Thereafter, by using halobenzazepine (SCH23390) (D1 dopamine receptor antagonist) and sulpiride (D2 receptor dopamine receptor antagonist), the role of dopamine receptors in STZ-induced depression was studied. The one-way analysis of variance technique, Tukey’s range test, and *t*-test were used to analyze the data. The P-value less than 0.05 was regarded as statistically significant.

**Results:**

Our study showed that STZ at doses of 30 and 60 mg/kg, two weeks after injection, caused prolonged immobility in FST, indicating depressive-like behavior (P<0.05 and P<0.01, respectively). SCH23390 (0.001 mg/mL/kg) and sulpiride (0.1 mg/mL/kg) did not change the variables of depression in animals that received STZ (at doses of 30 and 60 mg/mL/kg) two weeks before (P>0.05).

**Conclusion:**

According to our study, STZ has a depressive-like behavior two weeks after injection, and dopamine receptors do not play a role in depression associated with STZ use.

## Introduction

Diabetes is one of the most common endocrine diseases characterized by hyperglycemia induced by an absolute or a relative insulin deficiency or an insulin function deficiency [1]. The International Diabetes Federation (IDF) has predicted that the number of people with diabetes will reach up to 592 million in less than 25 years from now [2, 3]. Diabetes also causes complications such as retinopathy, nephropathy, neuropathy, stroke, as well as cardiovascular ones. Also, the disease causes cognitive impairments in learning, memory, mood flexibility, and especially depression [1, 4]. Diabetes mellitus is a risk factor for depression. According to studies, the likelihood of depression in diabetic patients is about 30% [5]. Diabetes and depression, together, increase the risk of complications, reduce the quality of life, reduce physical activity, and increase treatment costs and death rate [6, 7]. Several attempts have been made to treat and prevent the symptoms of depression associated with diabetes. One such attempt is the use of drugs to prevent depression associated with diabetes; however, the drugs have some limitations, and they lead to complications such as hypoglycemic effects, the long-term onset of therapeutic effects, and other complications [8-11]. Studies conducted with the forced swim test (FST) showed that the streptozotocin (STZ)-induced diabetes brings about depressive-like behavior in diabetic rats by increasing the immobility time in these rodents [12, 13].

Although the mechanism of diabetes-induced brain damage is not clearly identified, studies have shown that factors such as decreased insulin signaling, changes in glucose levels [14, 15], and changes in neurotransmitters such as dopamine play a role in mood and behavioral activity [16-18].

Dopamine is one of the most important neurotransmitters of the central nervous system that exerts its effects through special membrane receptors, including D1-like receptors (including D1 and D5) and D2-like receptors (including D2, D3, and D4). In the nervous system of mammals, the activators of the dopamine receptors play a role in regulating mood, motivation, reward, and motor function. These receptors are distributed in areas of the brain that are involved in actions such as emotion regulation, reward, motivation, pleasure, and memory [19-21]. Studies on diabetic and experimental diabetes have shown that diabetes and depression reduce dopamine secretion and the dopamine system activity in the brain, as well as upregulate dopamine receptors, possibly due to decreased dopamine synthesis and its retrograde signaling in the brain. Thereby disrupting the functions associated with dopamine [16, 18, 22, 23] in such a way that the substances that increase dopamine have antidepressant effects and reduce the immobility behavior in FST. The substances that reduce dopamine cause restlessness and depression [24, 25].

Considering the fact that perceptual problems, especially depression, seem to become one of the most important issues associated with diabetes in the future and the fact that studies have shown that various factors, including the changes in neurotransmitters such as dopamine, play a role in diabetes-induced depression, we aimed to study the role of dopamine receptors in STZ-induced depressive-like behavior using FST.

## Materials and Methods

### 1. Animals

This study was performed on 56 Wistar male rats weighing 230 to 250 g brought from the animal room of the Institute of Cognitive Sciences. The animals were given ad libitum access to food and water in a vivarium with a 12:12 hour light/dark cycle at 22 ± 2°C, and in groups of 3 to 4 per cage. There were seven rats in each study group, and each animal was used only once.

### 2. Forced Swim Test

FST is one of the animal models for studying depression that has been used since 1977. This test is performed by using a cylindrical chamber with the height of 40 cm and diameter of 15 cm, and that was filled with water to the height of 30 cm. The duration of the three movements of climbing, swimming, and immobility were measured in which the increase in the animal’s total immobility time is considered as a depressive-like behavior, which is similar to that of the human depression pattern [11, 12]. For the pretest and the test, the cylinder containers were filled with water to the height of 30 cm (at 22°C ± 2°C).

The water was replaced after use for each animal. FST consisted of 2 pretest and test steps. To carry out the pretest, each rat was made to swim in the cylinder for a period of 15 minutes. After 24 hours, under the same condition of forced swimming, the test was carried out for 6 minutes, in the last 5 minutes of which the time for climbing, swimming, and immobility was measured. The rising behavior included the upright movement of the animal alongside the swimming cylinder. The animal was swimming horizontally and was motionless when it was immobile in water with the least necessary movements to keep its head above the water. After testing, the animal was gently taken out of the water, dried with a towel, and placed back in the cage [26].

### 3. Drugs

The drugs used in this study consisted of STZ at doses of 30 and 60 mg/mL/kg, SCH23390 (R-(+)-7-chloro-8-hydroxy-3-methyl-1-phenyl-2,3,4,5-tetrahydro-1H-3-benzazepine hydrochloride as a dopamine D1 receptor antagonist, at a dose of 0.001 mg/mL/kg), and sulpiride (dopamine D2 receptor antagonist, at a dose of 0.1 mg/mL/kg). All the drugs were obtained from Sigma-Aldrich, St. Louis, MO, USA. The STZ and SCH23390 were dissolved in a solution of 0.9% saline immediately before injection, whereas sulpiride was dissolved in 1 drop of acetic acid. The control groups received saline or vehicle (1 mL/kg). All drugs were injected via intraperitoneal (IP) route here and at all subsequent occurrences.

### 4. Inducing Diabetes

STZ was used to induce diabetes and depression at doses of 30 and 60 mg/kg body weight in a cold physiological saline solution as a single dose and IP. Blood glucose was measured using a glucometer (Elegans, Germany) 3 days after the injection, and rats with blood glucose levels of 250 mg/dL or higher were considered as diabetic [27].

### 5. Experimental Design

#### 5.1. Effects of SCH23390 and Sulpiride on Immobility Time in FST

For this experiment, 3 groups of animals were used: animals of the first group received normal saline (1 mL/kg), those of the second group received SCH23390 (0.001 mg/mL/kg) 15 minutes before FST, and those of the third group received sulpiride (0.1 mg/mL/kg) 30 minutes before FST as IP. This experiment was designed to investigate the effect of SCH23390 and sulpiride on immobility time in FST.

#### 5.2. Effects of STZ 30 mg/mL/kg on Immobility Time in the Presence and Absence of SCH23390 and Sulpiride

For this experiment, four groups of animals were used: the first group received normal saline (1 mL/kg), whereas the second, third, and fourth groups received STZ (30 mg/mL/kg).

After 14 days, the animals of the first and second groups received normal saline (1 mL/kg), those of the third group received SCH23390 (0.001 mg/mL/kg) 15 minutes before FST, and finally those of the fourth group received sulpiride (0.1 mg/mL/kg) as IP 30 minutes before FST. This experiment was designed to investigate the effect of SCH23390 and sulpiride on the long-term immobility induced by STZ at a dose of 30 mg/mL/kg.

#### 5.3. Effects of STZ at a Dose of 60 mg/mL/kg on Immobility Time in the Presence and Absence of SCH23390 and Sulpiride

For this experiment, four groups of animals were used: the animals of the first group received normal saline (1 mL/kg), and those of the second, third, and fourth groups received STZ (60 mg/mL/kg).

After 14 days, the animals of the first and second groups received normal saline (1 mL/kg), those of the third group received SCH23390 (0.001 mg/mL/kg) 15 minutes before FST, and those of the fourth group received sulpiride (0.1 mg/mL/kg) as IP 30 minutes before FST. This experiment was designed to investigate the effect of SCH23390 and sulpiride on the long-term immobility induced by STZ at a dose of 60 mg/mL/kg.

#### 6. Statistical Analysis

All data were expressed as mean ± SEM. (standard error of mean). One-way analysis of variance (ANOVA) technique, Tukey’s range, and *t*-test were used to analyze the data. Values at P-value<0.05 were considered as statistically significant. SPSS software version 19 was used for data analysis.

## Results

### Effects of SCH23390 and Sulpiride on Immobility Time in FST

**Figure-1:**
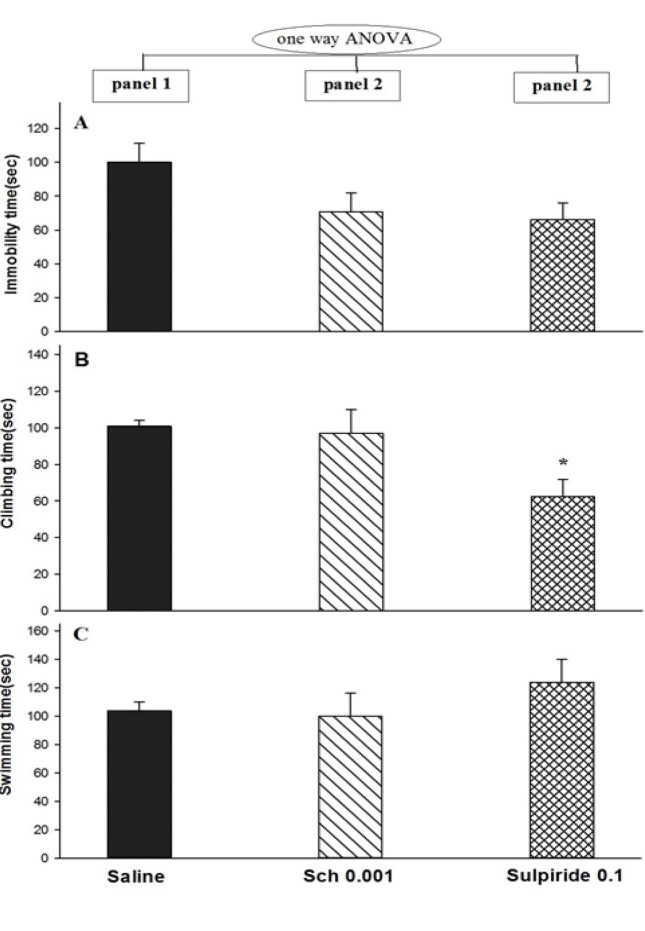
Effects of SCH23390 and sulpiride on immobility (panels 2A and 3A), climbing (panels 2B and 3B), and swimming (panels 2C and 3C). Three groups of animals were used: the animals of the first group received normal saline (1 mL/kg), those of the second group received SCH23390 (0.001 mg/mL/kg), and those of the third group received sulpiride (0.1 mg/mL/kg). Values are expressed as mean ±standard error of mean (SEM). (n=7 in each group).

One-way ANOVA and Tukey’s test showed that SCH23390 and sulpiride did not alter immobility (F_2,18_=0.855; P>0.05; Figure-1, panels 2 and 3A) and swimming (F_2,18_=2.953; P>0.05; Figure-1, panels 2 and 3C), whereas sulpiride decreased climbing (F_2,18_=5.170; P<0.05; Figure-1, panel 3B).

### Effects of STZ at a Dose of 30 mg/mL/kg on Immobility Time in the Presence and Absence of SCH23390 and Sulpiride

**Figure-2:**
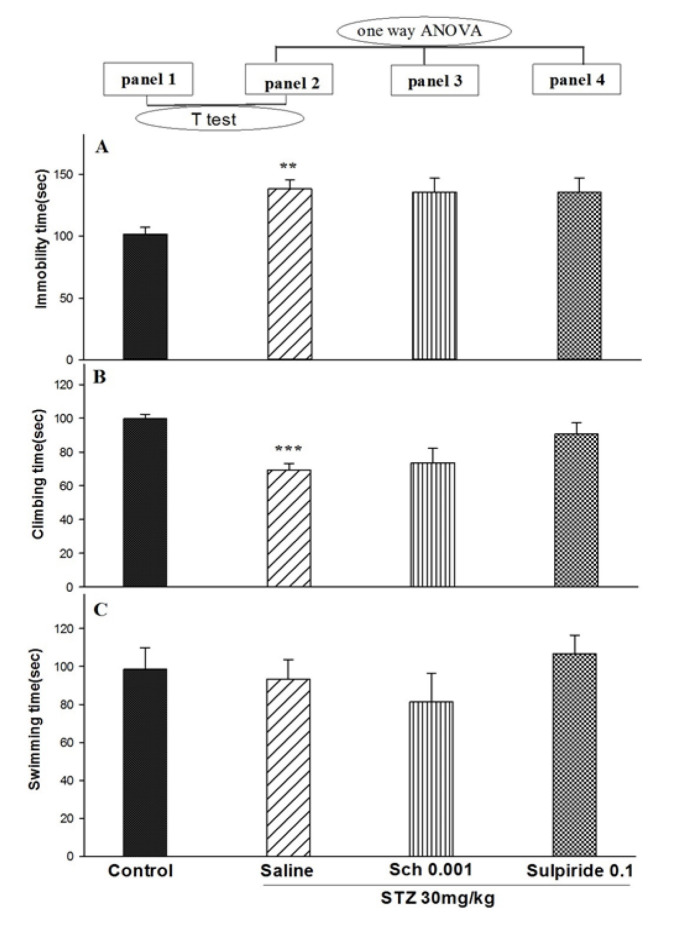
Effects of the dose of 30 mg/mL kg STZ on immobility (panels 2A, 3A, and 4A), climbing (panels 2B, 3B, and 4B), and swimming (panels 2C, 3C, and 4C) in the presence and absence of SCH 23390 or sulpiride. A total of 4 groups of animals were used: the animals of the first group received normal saline (1 mL/kg), and those of the second, third, and fourth groups received STZ (30 mg/mL/kg). After 14 days, the animals of the first and second groups received normal saline (1 mL/kg), those of the third group received SCH23390 (0.001 mg/mL/kg), and those of the fourth group received sulpiride (0.1 mg/mL/kg). Values are expressed as mean ± standard error of mean (SEM). (n=7 in each group). **P<0.01 and ***P<0.001 compared with the control group in panel 1.

The *t*-test showed that STZ at a dose of 30 mg/mL/kg increased immobility (*t*=3.959; P<0.01; Figure-2, panels 2A), whereas it decreased climbing (*t*_12_=6.776; P<0.001; Figure-2, panels 2B); it did not alter swimming (*t*_12_=0.346; P>0.05; Figure-2, panel 2C). One-way ANOVA and Tukey’s range test showed that SCH23390 and sulpiride did not alter STZ (30 mg/mL/kg) responses for immobility (F_2,18_=0.22; P>0.05; Figure-2, panels 3 and 4A); climbing (F_2,18_=2.868; P>0.05; Figure-2, panels 3 and 4B), and swimming (F_2,18_=1.126; P<0.05; Figure-2, panels 3 and 4C).

### Effects of STZ at a Dose of 60 mg/mL/kg on Immobility Time in the Presence and Absence of SCH23390 and Sulpiride

**Figure-3:**
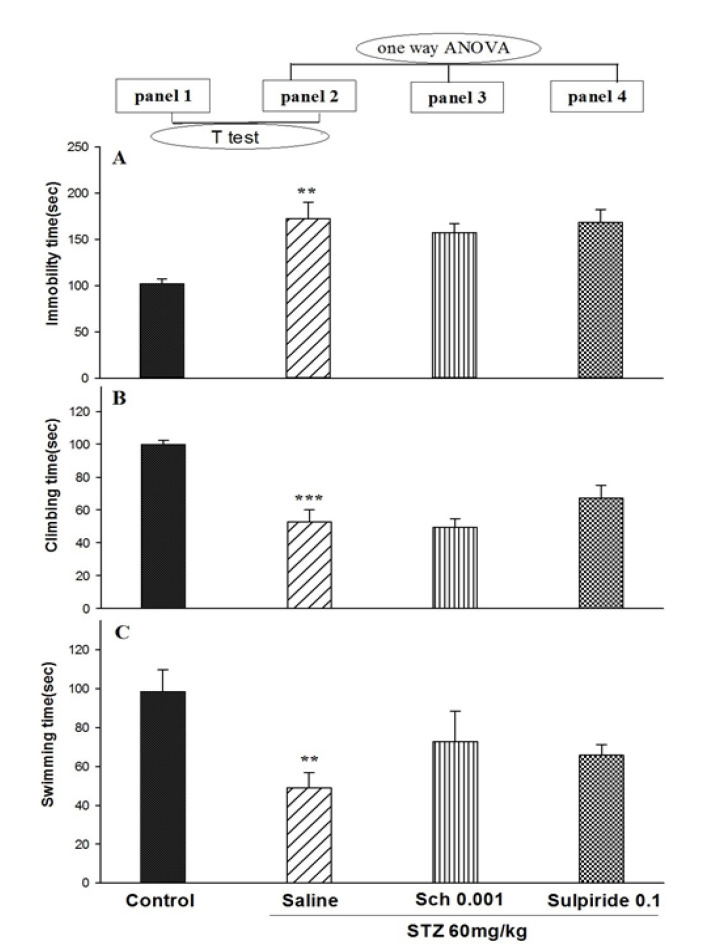
Effects of STZ 60 mg/mL/kg on immobility (panels 1A, 2A, and 3A), climbing (panels 1B, 2B, and 3B), and swimming (panels 1C, 2C, and 3C) in the presence and absence of SCH 23390 or sulpiride. A total of 4 groups of animals were used: the animals of the first group received normal saline (1 mL/kg), and those of the second, third, and fourth groups received STZ (30 mg/mL/kg). After 14 days, the animals of the first and second groups received normal saline (1 mL/kg), those of the third group received SCH23390 (0.001 mg/mL/kg), and those of the fourth group received sulpiride (0.1 mg/mL/kg). Values are expressed as mean ± standard error of mean (SEM). (n=7 in each group). **P<0.01 and ***P<0.001 compared with the control group in panel 1.

The *t*-test showed that STZ at a dose of 60 mg/mL/kg increased immobility (*t*_12_=3.765; P<0.01; Figure-3, panels 2A), whereas it decreased climbing (*t*_12_=6.120; P<.001; Figure-3, panels 2B) and swimming (*t*_12_=3.633; P<0.01; Figure-3, panel 2C). One-way ANOVA and Tukey’s test showed that SCH23390 and sulpiride did not alter STZ (60 mg/mL/kg) responses for immobility (F_2,18_=0.305; P>0.05; Figure-3, panels 3 and 4A), climbing (F_2,18_=1.918; P>0.05; Figure-3, panels 3 and 4B), and swimming (F_2,18_=1.349; P>0.05; Figure-3, panels 3 and 4C).

## Discussion

Our study showed that STZ at doses of 30 and 60 mg/kg for two weeks after the injection caused a long-term immobility in FST, which means depressive-like behavior in diabetic rodents. STZ at the dose of 60 mg/kg reduced climbing and swimming activities, whereas STZ at a dose of 30 mg/kg reduced climbing activity. It also showed that three days after the injection of STZ at the dose of 60 mg/kg, the blood glucose levels increased (as the most important sign of diabetes) and diabetes was induced. However, no change in blood glucose was observed at the dose of 30 mg/kg. Our results also showed that dopamine receptors blockade with SCH23390 and sulpiride did not alter the STZ-induced depressive-like behavior. The despair-based behavioral tests, including FST, are the models that are widely used for studying depressive-like behavior. This test is designed in such a way that when the rat is forced to swim in a restricted area, it stops trying after a while and stays still, indicating lowered mood or hopelessness [28, 12, 10]. In this study, consistent with other studies, STZ has been shown to increase immobility time, which reflects the depressive-like behavior caused by STZ. Although studies have shown that three and four weeks after STZ injection, immobility activity in FST increases [13, 15, 29], some other studies, such as ours, have shown an increase in immobility two weeks after STZ injection [14, 12].

Studies show that the risk of depression in people with diabetes is 2 to 3 times more than that in others [26, 5]. Depression in diabetes may be due to several factors, the most important of which are the changes in neurotransmitters, including dopamine. Dopamine is involved in activities such as pleasure, memory, and reward through receptors that are scattered all over the brain [21, 20]. Studies show that diabetes and induced diabetes can change the concentration of dopamine, its metabolites, and dopamine receptors in the brain. Diabetes has been shown to reduce dopamine in the brain and plasma (as an indicator of central dopamine levels), as well as reduce the dopamine transporter [18, 16, 17, 30].

It is also shown that in the incidence of depression and diabetes mellitus, mRNA and dopamine receptors undergo an upregulation, which indicates a decrease in dopamine transmission [25, 14]. Also, pharmacological studies have shown that the experimentally induced diabetes decreases the tyrosine and tyrosine hydroxylase levels in the brain. Dopamine is made from tyrosine by tyrosine hydroxylase [18, 31, 16]. As a result, the changes in dopamine and its receptors decrease dopamine signaling and its related functions such as motivation, attention, pleasure, and reward.

Although some studies have shown that the dopamine receptors in diabetes undergo an upregulation and are likely to be involved in diabetes-induced depression [22, 23], our study showed that the dopamine receptor blockade did not significantly change the depressive-like behavior induced by STZ. In other words, they were not involved in the depression caused by STZ. One reason for this might be the fact that, in diabetes, the levels of other neurotransmitters involved in mood regulation decrease. As reported in the studies involving diabetic and STZ-induced diabetic patients, the activity of serotonin, as one of the most important neurotransmitters involved in regulating mood and behavior, is impaired in the brain of people with diabetes, and the serotonin level in the brain and plasma significantly increase because of the reduced biosynthesis of serotonin as a result of decreased levels of L-tryptophan in the plasma and brain. This is associated with a decrease in the activity of tryptophan hydroxylase and the long-term rate-limiting effect of the enzyme [14, 32]. Also, some studies have reported that the levels of other neurotransmitters such as norepinephrine decrease in diabetes and depression [33, 16].

The interaction among the neurotransmitter systems can be considered as another reason for depression. It is known that there is a positive relation between dopamine and serotonergic systems, and they are related in such a way that an increase in serotonin and norepinephrine causes a similar increase in dopamine signaling. On the other hand, any defect and dopamine deficiency in diseases such as Parkinson disease can cause a deficiency and a decrease in the serotonergic system and serotonin transporter, which can be one of the causes of depression [25, 34]. The low dose of the antagonist used for dopamine receptors can also be considered as another contributing factor for the depression.

## Conclusion

Our study showed that STZ at doses of 30 mg/kg and 60 mg/kg, 14 days after injection, caused an indication of depressive-like behavior. SCH23390 and sulpiride, as D1 and D2 dopamine receptor antagonists, did not alter the STZ-induced depressive-like behavior, and they did not play any significant role in this regard. However, more studies such as molecular studies are needed to examine the role of dopamine receptors in depression induced by STZ.

## Conflict of Interests

None declared.
